# Letter from Dr. Haven, St. Louis, Mo.

**Published:** 1848-10

**Authors:** Jas. H. Haven


### LETTER FROM DR. HAVEN.

St. Louis, Aug. 26, 1848.

*Dear Sir:—*

I have at your request made an examination of the substance you said was used for plugging decayed teeth, called, I think, Hill's Stopping.

The small quantity at my disposal, did not allow of an exact quantitative analysis, the following approximation may answer your purpose.

This substance is in the form of rolls about the size of a pipe stem, of a grayish white color, easily cut with a knife, and may be indented with the finger nail; is not easily pulverised, softens in hot water so as to become plastic, exposed to the heat of a spirit lamp on a platina plate, it does not melt so as to change its form, and burns with a fuliginous flame. It is not attacked by alkalies in solution, nor readily by dilute acids when in mass, is not soluble in alcohol, but a portion is, with great facility, dissolved by the essence of turpentine, and also by chloroform, which solvents leave a heavy insoluble residue. 50 cent. grain treated by chloroform left 11c. gr. of a granular white powder, much insoluble matter remaining suspended in the solution.—The heavy powder is completely soluble in dilute nitric acid, with effervescence, consequently containing little or no silex, this nitric solution gave me the reactions of lime and magnesia by the application of proper tests. The portion dissolved in chloroform in its turbid state applied to the skin soon dries and being very adhesive—this solution was precipitated by alcohol, which throws down a curdy mass, and when this is washed with hot water, it may be united into a mass resembling the original compound, and weighed 48½c. gr.—this was calcined on a platina capsule, and yielded a very white friable mass, which communicated to water strong alkaline properties, nitric acid was added to this ashes and evaporated to expel the excess of acid, and re-dissolved in water, a few brown flakes appeared and the solution was slightly colored by iron, when concentrated this nitric solution yielded by cooling a mass of needle chrystals, highly deliquesent. The solution of this salt is abundantly precipitated by the oxalate of ammonia, and a concentrated solution of sulphuric acid—this acid does not cause a precipitation when very dilute.

This nitrate was converted into sulphate by sulph. acid q. s., and heated to incipient redness, and treated with boiling water, much insoluble sulphate remained and the clear liquid was precipitated by oxalate of ammonia and filtered, the solution then gave another white precipitate by the phosphate of soda and ammonia, and the peculiar streak on glass by friction indicative of magnesia.

Again, I calcined 50 c. gr. of the fresh material, which gave 40 c. gr. of an earthy ash—loss by combustion 10 c. gr. The 40 c. gr. of ashes, treated by sulphuric acid effervesces and becomes somewhat gelatinous, in the manner of the ashes of bones. I did not at the time examine for phosporic acid, which I might have found. The sulphate washed and dried, weighed 41 c. gr. sulphate of lime, an additional quantity of lime was obtained by oxalate of ammonia—then the clear solution treated with the phosphate of soda and ammonia, gave a chrystaline deposit leaving a trace on glass, soluble in dilute acetic acid, which converted into sulphate and dried, weighed 07 c. gr. sulphate magnesia.

Although the above is a very imperfect analysis, it is evident that this material contains 80 per cent. of earthy matter, consisting of salts of lime and magnesia, probably as phosphates and carbonates, and 20 per cent. of an organic substance having the properties of gutta percha.

Should you make the experiment of incorporating 4 parts bone ash in powder, with 1 part gutta percha dissolved in chloroform, or ess. tereb., and allow the mass to dry, I think you will obtain a similar, if not an identical substance with this under examination, for the bone ash contains phosphate lime with traces of magnesia, and also some carbonates, and will give the reactions as those above. The gutta percha would in a great measure, shield these earths from the action of dilute acids when in a mass, and would be soluble in ess. tereb. and chloroform, and precipitated by alcohol, and would act in the flame in the same way. Yours, respectfully, JAS. H. HAVEN.

